# Jaundice and Higher Procalcitonin Level Revealing a Small-Cell Lung Cancer With Pancreatic Metastasis: A Case Report From Eastern Morocco

**DOI:** 10.7759/cureus.58041

**Published:** 2024-04-11

**Authors:** Hind Chibani, Soufia El Ouardani, Mouhsine Omari, Karich Nassira, Ouissam Al Jarroudi, Hanane Hadj Kacem, Sami Aziz Brahmi, Amal Bennani, Said Afqir

**Affiliations:** 1 Medical Oncology, Mohammed VI University Hospital, Oujda, MAR; 2 Medical Oncology, Faculty of Medicine and Pharmacy, Mohammed First University, Oujda, MAR; 3 Pathology, Mohammed VI University Hospital, Oujda, MAR; 4 Pathology, Faculty of Medicine and Pharmacy, Mohammed First University, Oujda, MAR; 5 Radiology, Mohammed VI University Hospital, Oujda, MAR; 6 Radiology, Faculty of Medicine and Pharmacy, Mohammed First University, Oujda, MAR

**Keywords:** metastatic non-small-cell lung cancer, jaundice, platinum-based chemotherapy, procalcitonin, pancreatic metastasis

## Abstract

Small-cell lung cancer (SCLC) is highly aggressive, with a severe tendency for metastasis. Pancreatic metastasis in SCLC is uncommon, also jaundice as a major symptom of small-cell lung cancer is even rarer. The diagnosis of pancreatic metastasis is a real challenge for the medical team, it relies on both radiological and pathological details. We report a case of a 58-year-old male admitted for SCLC with pancreatic metastasis and a higher level of procalcitonin. He received platinum-based chemotherapy with a swell response. The focus of this study will be on the characteristics of pancreatic metastasis, along with their diagnosis and treatment approaches. Procalcitonin as a paraneoplastic syndrome will also be discussed in this study.

## Introduction

Lung cancer stands as a global health issue with severe mortality rates, strongly linked to smoking. Unfortunately, it is the leading cause of cancer-related deaths worldwide [1]. In Morocco lung cancer is the first male cancer according to the Casablanca register [2]. There are two components of lung cancer which are non-small-cell lung cancer (NSCLC) and small-cell lung cancer (SCLC). There are no data on SCLC in Morocco. We have found only one retrospective study published in the Oriental region of Morocco, which accounts for approximately 12.47% of all cancers [3].

The sites where metastasis occurs most often in the case of SCLC are brain, bones, liver, adrenal gland, contralateral lung, and distant lymph nodes [4]. Other organs are rare, such as pancreatic metastasis [5]. Only a few cases are listed in the literature. In this study, we report a case of a 58-year-old male who presented with SCLC with pancreatic metastasis and a higher level of procalcitonin.

## Case presentation

**Figure 1:**
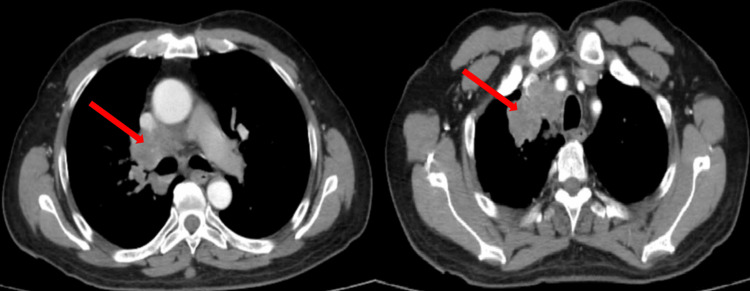
CT chest sections of the patient. Pulmonary tumoral process of the apicoventral segment of the right upper lobe of 45×42×42 mm in large axes (arrows), with homolateral medial ganglionic magma of 75 mm in his posterior-anterior diameter with bilateral pulmonary micronodules of suspicious appearance.

**Figure 2:**
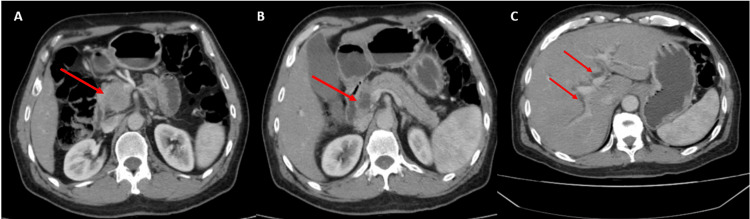
CT abdominal sections of the patient. Pancreatic head processus measuring 51×48×35 mm in large axes (arrows) which can be primitive or secondary (A) responsible for biductal biliary expansion of Wirsung at 4 mm and the main biliary tract at 12.5 mm (B) with dilation of the intra-hepatic biliary tract (C).

We report a case of a 58-year-old male, a heavy smoker 47 pack-years, he was admitted to the emergency due to abdominal pain and cough evolving for six months, with worsening three days earlier by the appearance of acute jaundice. A blood test was done urgently, and the results of the infectious assessment and cholestasis were disturbed. The patient has benefited from a thoracoabdominal CT scan in favor of a pulmonary tumor process of the apicoventral segment of the right upper lobe with homolateral medial lymph node magma, associated with a tumor process of the pancreas responsible for bicanalar dilatation of Wirsung and main bile duct, with dilatation of intra-hepatic bile duct and gallbladder (Figure 1 and Figures 2A-2C).

**Figure 3:**
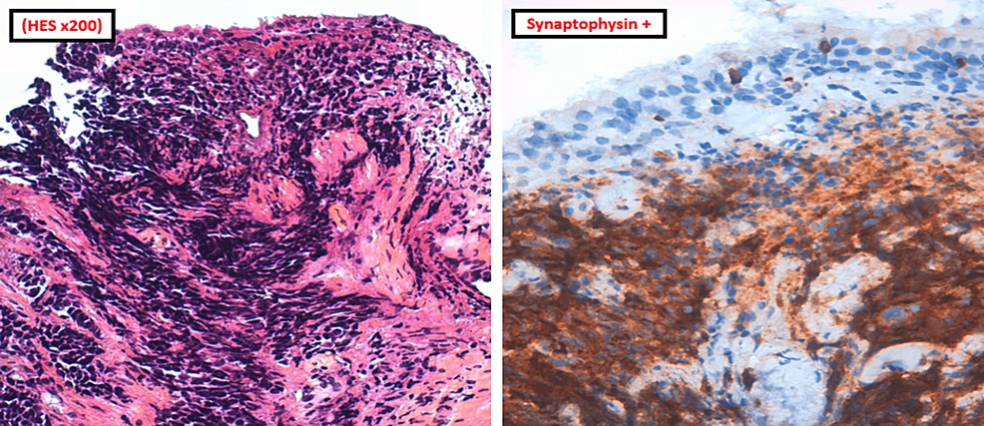
Tumor proliferation of bronchial mucosa (HES×200) was positive for synaptophysin. Bronchial mucosa site of tumor proliferation, largely crushed, made of small-to-medium-sized cells with nuclei with mottled and dense chromatin. This tumor proliferation is positive for synaptophysin.

**Figure 4:**
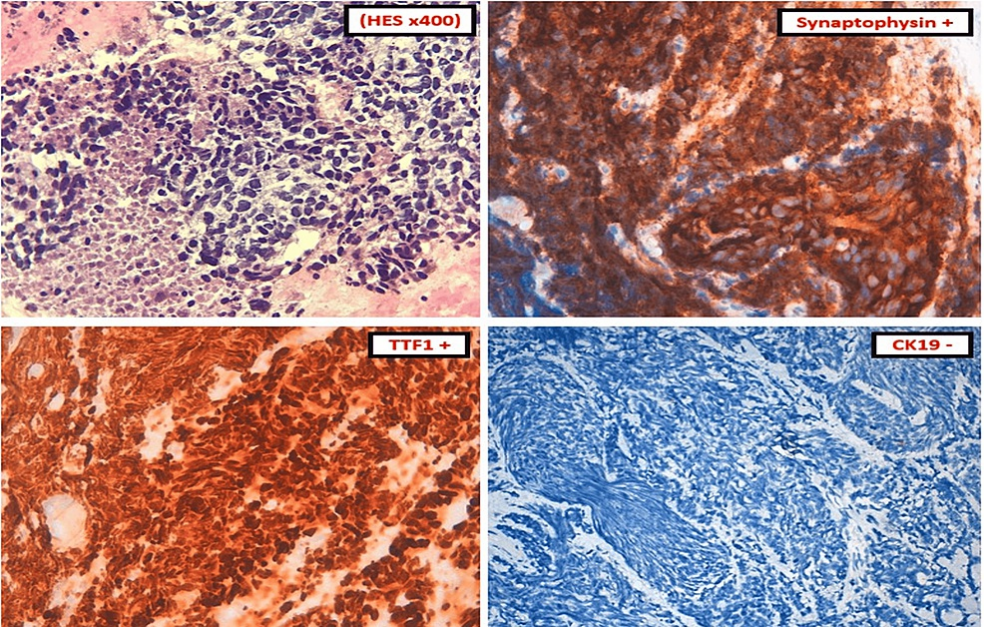
Pancreatic immunochemistry of the patient. Pancreatic cytopuncture objectified the presence of focal necrotic tumor proliferation, cytonuclear characteristics of which recall bronchial tumor proliferation. Immunohistochemical study confirmed lung origin by TTF1 and eliminated biliopancreatic.

As our patient had angiocholitis, he underwent his first endoscopic ultrasound-guided fine needle aspiration and insertion of a plastic stent. the patient also received antibiotic therapy. A few days later, bronchial fibroscopy and biopsy were performed on the patient and a brain CT scan as part of the extension assessment. The outcomes of both needle aspiration and bronchial biopsy were in favor of small-cell lung cancer in a way that bronchial mucosa was a site of tumor proliferation, largely crushed, made of small-to-medium-sized cells with nuclei with mottled and dense chromatin. This tumor proliferation is positive for synaptophysin (Figure 3). Also, pancreatic cyst puncture objectified the presence of focal necrotic tumor proliferation, the cytonuclear characteristics of which recall bronchial tumor proliferation. An immunohistochemical study confirmed lung origin based on positive staining for TTF1 and negative staining for CK19, thus ruling out a bilio-pancreatic origin (Figure 4).

Clinical and biological improvement as well as normalization of hepatic functions were the hallmarks of the evolution. Despite the persistently high rate of procalcitonin, it was in the range of 9.78 ng/L, likewise, a full infectious assessment was done, and it was found to be negative including a negative blood culture test (Table 1). At list we had retained the diagnosis of small-cell lung cancer metastatic in pancreas with a paraneoplastic higher level of procalcitonin.

**Table 1:** Results of blood investigations before and after treatment.

Blood test	At diagnosis (before treatment)	After drainage and antibiotic therapy	After chemotherapy
Total bilirubin (mg/L)	80	9.3	4.3
Conjugated bilirubin (mg/L)	56	6	3
Aspartate transferase (AST) UI/L	131	22	14
Alanine aminotransferase (AST) UI/L	131	22	12
Gamma-glutamyl transpeptidase (GGT) UI/L	650	138	132
C-reactive protein (CRP) mg/L	100	12.52	11
Procalcitonin (PCT) ng/L	22	9.78	0.22

This case was discussed in a multidisciplinary meeting consultation. The general condition was preserved as were the hepatic and renal functions, and due to the unavailability of immunotherapy in our hospital, the patient received cisplatin 80 mg/m^2^ (day one) with etoposide 100 mg/m^2^ (from day one to day three) every three weeks. The treatment was well-tolerated with the exception of grade 1 nausea and vomiting.

After three courses of chemotherapy, the evaluation was considerable. The general state has greatly enhanced. It was marked by a decrease in procalcitonin rate. Furthermore, a CT brain-neck chest-abdomen-pelvis showed a partial response with complete resolution of the pancreatic process (Table 1).

## Discussion

The metastatic potential of SCLC is higher than other tumors, and it was long misunderstood. It was explained by diffusing through lymphatic and hematological spread. Fortunately, developmental biology has solved the mess by supporting new theories, mainly genomic alterations, such as inactivation of suppressor gene tumors (P53/Rb1), circulating tumor cells, and epithelial-to-mesenchymal transition [6].

In line with the literature, pancreatic metastasis in SCLC seems to be a rare localization. The case series of Niu et al. includes 193 patients with atypical metastasis out of 2872 patients diagnosed with NSCLC. The frequency of pancreatic localization was 0.59%. Pursuant to the same study, survival was worse when unusual metastasis was found [7].

In a retrospective analysis of 1659 cases, 33 patients were diagnosed with pancreatic metastasis and SCLC as primary tumor was identified among 18 patients [8]. Conjointly in a literature review of Gonlugur et al., pancreatic metastasis occurs between 1.6% and 10.6% in autopsy studies. Jaundice in small-cell lung cancer is due to the liver tract, which is narrowed by metastasis of lymph nodes in the porta hepatis, pancreas, or hepatic parenchyma [9].

We spotted that isolated imaging is not sufficient to diagnose pancreatic metastasis. Therefore, endoscopic ultrasound-guided fine needle aspiration is mostly required to confirm the primary or secondary origin of pancreatic injury [10]. As well as this dilemma is avoided by completing a whole immunochemistry panel. The most needed in practice are neuroendocrine markers, such as chromogranin, synaptophysin, and CD56; another new neuroendocrine marker is INSM1 [11].

The historical treatment of SCLC is platinum-based chemotherapy, as shown in clinical trials and meta-analyses, the association of cisplatin and etoposide significantly improved survival [12,13]. Most recently, the basic treatment of SCLC is the addition of programmed cell death protein-1 (PD-1) and programmed death ligand-1 (PDL-1) inhibitors to conventional chemotherapy, atezolizumab and durvalumab are among the key drugs in this approach [14]. Also, according to the European Society for Medical Oncology (ESMO) guidelines, an anti-PDL-1 inhibitor in combination with platinum and etoposide chemotherapy is the reference treatment for extended stages.

Another point that remains important to discuss in the present case is the paraneoplastic higher level of procalcitonin (PCT). PCT is known as an infectious biomarker. It is made up of 116 amino acids that are derived from pre-PCT and encoded by the calcitonin 1 gene on chromosome 11. Thyroid C cells are the only ones responsible for producing mature calcitonin, although other neuroendocrine cells also contribute to a lesser extent. Besides, PCT can be elevated in thyroid carcinoma and lung cancer due to proinflammatory or hypoxic situations of tumor microenvironment. Only a few studies have demonstrated the impact of procalcitonin in cancer prognostics. The study by Kajikawa et al., which includes 51 patients suffering from NSCLC, suggested in conclusion that a high serum level of PCT is associated with worse overall survival [15]. Concurrently a study of 147 patients with lung cancer suggested that the PCT level was higher in SCLC than in adenocarcinoma [16].

In the present case, the patient was treated only with chemotherapy due to the unavailability of immunotherapy in our hospital. Both clinical and radiological responses were striking. He is still alive with a good performance status, undergoing his chemotherapy courses.

## Conclusions

Obstructive jaundice as a major clinical sign revelator of SCLC is rare. This serves as a reminder to clinicians that jaundice due to pancreatic tumor should always be well investigated considering that not all pancreatic processes are primary tumors; therefore, not missing a pertinent treatment. Also, we need to encourage more testing of procalcitonin serum levels to try to find a consistent theory that links PCT to SCLC and maybe grows into a prognostic factor.
